# Lively contributions: 156 years after the death of Jan Evangelista Purkyně

**DOI:** 10.1055/s-0045-1809402

**Published:** 2025-06-20

**Authors:** Catarina Dantas Corrêa, Camila Emi Fujiwara Murakami, Isabelle Pastor Bandeira, Carlos Roberto M. Rieder, Hélio Afonso Ghizoni Teive

**Affiliations:** 1 Universidade Federal do Paraná, Departamento de Clínica Médica, Serviço de Neurologia, Curitiba PR, Brazil.; 2 Universidade Federal de Ciências da Saúde de Porto Alegre, Departamento de Neurologia, Porto Alegre RS, Brazil.

**Keywords:** Jan Evangelista Purkyně, History of Medicine, Neurology

## Abstract

Jan Evangelista Purkyně, born on December 17, 1787, in Bohemia (now part of the Czech Republic), was a prominent scientist renowned for his discoveries in eye, brain, and heart physiology. To honor him 156 years after his death, the present article explores Purkyně's history, from the struggles of his youth to the main legacies he left in medicine, especially neurology.

## INTRODUCTION

**Figure 1:**
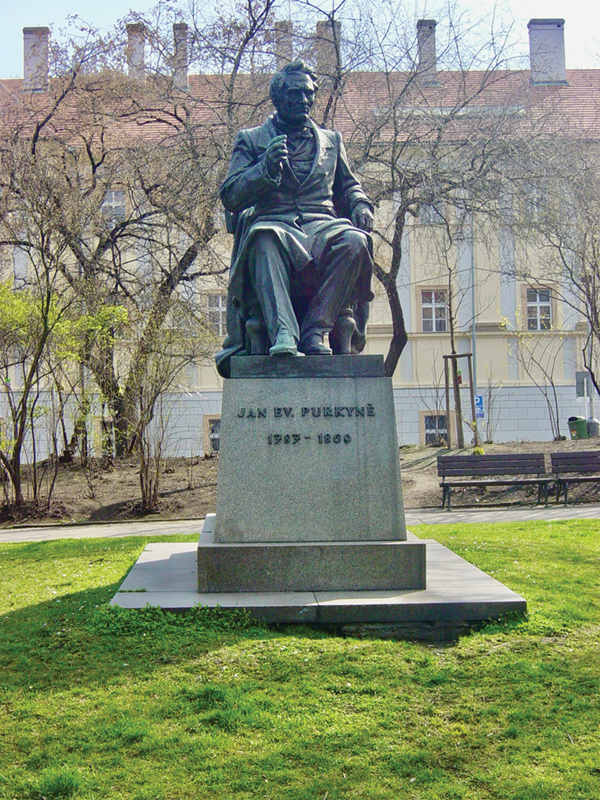
Jan Evangelista Purkyně (1787–1869).

Jan Evangelista Purkyně 1 2 3 4 (1787–1869) ( Figure 1 ) was a remarkable Czech scientist who was a professor of physiology in Wroclaw/Breslau, Poland, and later at the University of Prague. Purkyně made discoveries related to the structure and function of the eye, brain, and heart, which resulted in countless eponyms attributed to him. 1 2 3 4 5 In addition to working in the biological sciences, Purkyně translated poetry from German, Russian, and Polish into Czech, as an active Czech patriot, he contributed substantially to the construction of a strong national Czech identity. 5 The objective of the present work is to discuss the numerous contributions of this neuroscientist, in this year, which marks the 156th anniversary of his passing.

## BIOGRAPHICAL DATA

On December 17, 1787, Jan Evangelista Purkyně was born in Libochovice Castle, then Bohemia, part of the Austro-Hungarian Empire (now part of the Czech Republic). 1 2 3 4 5 6

When Purkyně was 6 years old, his father died, and the family was left without financial security. Nevertheless, he attended the local primary school in Libochovice, where, impressed by the young boy's intelligence, the local chaplain taught him more than the basic curriculum. By the age of 10, Purkyně was selected for the boys' choir of the Piarist monastery of Mikulov, where he completed his gymnasium schooling. In 1804, he entered the Piarist order at the Stará Voda monastery, not intending to become a priest, but to acquire advanced education. 1 2 3 4 5 6

Purkyně left the Piarist order in 1807 and moved to Prague to become a student at the Philosophical Institute of Universitas Carolo-Ferdinandea, where he attended physical science courses and worked as a private tutor in 1809 due to financial needs. At the end of 1812, he enrolled as a student at the University of Prague Medical School. These studies culminated with his dissertation, *Contributions to the Knowledge of Vision from the Subjective Point of View* , in 1818. 1 2 3

Between 1819 and 1822, Jan Purkyně worked as an anatomy instructor at the University of Prague Medical School. During this period, he unsuccessfully applied for professorships at universities within the Habsburg Empire. It was only after the support of Johann Nepomuk Rust (1775–1840), Professor of Physiology at Berlin University, and an endorsement letter from Karl Asmund Rudolphi (1771–1832), Professor of Anatomy and Physiology at Berlin University, that Purkyně was accepted, in 1823, as Professor of Physiology and Pathology at the Medical School of the Royal Prussian University of Breslau/Wroclaw. 1 2 3 4 5 Another possible important endorser of Purkyně's professorship was writer and philosopher Johann Wolfgang von Goethe, whose theories of color were supported by Purkyně's theses in the domain of color vision. 7 His nomination, however, was not well accepted by his fellow colleagues as he was a foreigner and did not have the German tittle of *Dozent* (university teacher). Gabriel Gustav Valentin (1810–1883) was Purkyně's main disciple. 1 2 3 4 5 7

Rudolphi, known as the “father of helminthology,” was one of Purkyně's greatest enthusiasts, and he would later become Purkyně's father-in-law, following his marriage to Julia Rudolphi, in 1827. The couple had four children. Their daughters died of cholera in early childhood, but their two sons, Emanuel, a naturalist, and Karel, a portrait painter, survived into adulthood. In 1835, Julia died of typhoid fever. 5

In November 1839, Purkyně founded the Wroclaw Institute of Physiology, which was dedicated to research and to furthering Czech and Polish cultures. He was a Czech nationalist in a then academic atmosphere dominated by the German language and culture. 7 In 1850, back in Prague, he accepted the position of Professor of Physiology at the Medical Faculty of Charles University (Prague). 1 2 3 4 5

Just one year after being honored by the Imperial Austrian Order of Leopold in 1868, awarded for merit and moral integrity, Purkyně passed away on July 28, 1869, in Prague. 1 2 3 4 5

Jan Purkyně's social, cultural and scientific achievements made him extremely famous during his late life and afterwards. Regardless, he neither sought nor expected fame, as shown in a statement Purkyně made in the year of his death: “... A hundred years hence perhaps only a few will know who Purkyně was. But that makes no difference. For indeed we do not know who discovered the plow, and yet it serves all humanity. The cause remains the same, but not the name, and that is the important thing.” 5

## CARDIOLOGICAL CONTRIBUTIONS

Although his primary focus was not on cardiology, Purkyně left a substantial legacy in distinct areas within this field. 6 8 In 1839, he observed, in the ventricular subendocardium of sheep hearts, a network of gray fibers extending to the papillary muscles and other fibrous trabeculae. 8 These fibers are now recognized as cardiomyocytes that specialize in conducting electrical signals from the His bundle to the contractile ventricular myocytes: the Purkyně fibers. This discovery elucidated the mechanisms of cardiac contraction and their crucial role in circulatory function. 4 6 8

Pharmacology was another significant field of Purkyně's work: he explored beyond the analysis of the color, smell, and taste of drugs (largely of vegetal origin) to investigate their therapeutic profiles in humans. 4 6 8 His experiments with digitalis leaf extract highlighted its effects and side effects (bradycardia, nausea, and blurred vision), challenging prevailing medical practices of the time and paving the way for more rigorous pharmacological studies. 5 6 8

## NEUROLOGICAL CONTRIBUTIONS

**Figure 2:**
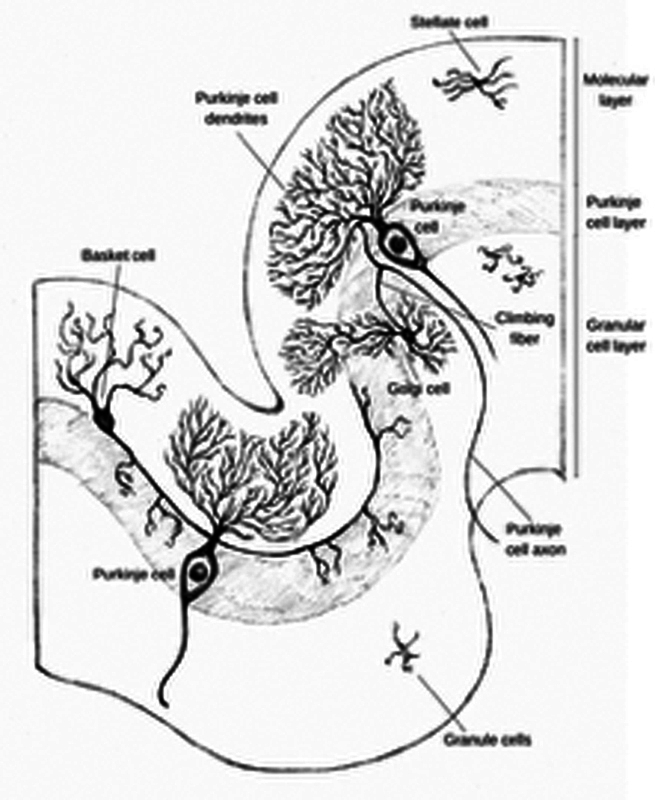
Cerebellar cortex with Purkyně cells (by Pedro Mansor, 2025).

Purkyně was a pioneer in nineteenth-century neuroscience, laying foundational groundwork through meticulous studies of the central nervous system. His neurohistological investigations comprehensively described various nerve cell types across different brain regions, including the cerebellar cortex ( Figures 2 3 ), hippocampus, and substantia nigra. 1 2 3 4 5 6 9 10 11 12

**Figure 3:**
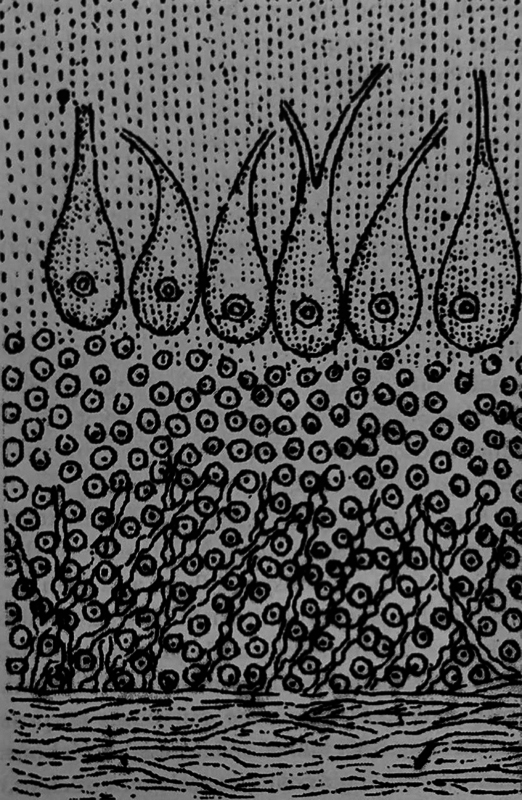
Purkyně's drawing of the cerebellar cortex, 1838. 7

Among the many eponyms attributed to the Czech physiologist, one of the most celebrated is the Purkyně cells, the largest neurons of the cerebellum. In 1837, during a lecture in Prague, Purkyně illustrated them and named them *ganglionic bodies* . 12 Purkyně researchers examined slices of sheep cerebellum fixed in alcohol in a series of experiments that would accurately describe each of the three layers of the cerebellar cortex. 12 13 Through these experiments, Purkyně attributed to the cerebellum the role of precision of motor movements. Seventy-four years later, in 1911, Cajal 14 recommended renaming the so-called ganglionic bodies after the author who first described them.

Beyond anatomical descriptions, Purkyně introduced significant functional concepts, notably distinguishing between ganglionic bodies and nerve fibers as generators and conductors of neural energy. 10 His studies on neuronal processes, including the initial characterization of dendrites, underscored his commitment to understanding the intricate relationship between neuronal structure and function. Purkyně's meticulous approach, aided by innovative tools such as the ocular micrometer, enabled precise measurements that furthered neuroscientific understanding. 10

Purkyně also dedicated himself to investigating the mechanisms of balance and vertigo, giving rise to Purkyně's law of vertigo. This law states that when one stops after rotating around the body-axis, the apparent motion of the surroundings changes from horizontal to vertical if the head is inclined toward the feet. 4 He proposed that the cerebellum was the main structure responsible for vertigo, which was later refuted by the description of the role of the vestibular system.

Purkyně's 1836 study on ependymal ciliary cells throughout the cerebral ventricles and subsequent observations of ciliary movements in brain cavities in 1858 also highlighted his multifaceted contributions to neurology 2 3 4 9 14 ( Table 1 ).

**Table 1:** Main contributions of Purkyně to neurology

Date	Discovery	Reference
1820–1827	Purkyně's law of vertigo	Purkyně, J. E. Über das Gleichgewicht der Flüssigkeit in den inneren Ohren der Säugethiere. *Archiv für Anatomie, Physiologie und Wissenschaftliche Medicin* , 1820. Purkyně J. Beyträge zur näheren Kenntniss des Schwindels aus heautognostischen Daten. *Medicinische Jahrbücher des kaiserlich-königlichen öesterreichischen Staates* , v. 6, p. 79–125, 1820.
1836	Description of ependymal ciliary cells along the brain ventricles	Purkyně, J. *Müllers Arch Anat Physiol* , v. 3, p. 289, 1836.
1837	Description of cerebellar cells, then named *ganglionic bodies* , now known as *Purkyně cells*	*Über Neuesten Untersuchungen* *aus der Nerven- und Hirn-Anatomie* (“About New Investigations on Nerves and Brain”)
1837	Description of the three layers of the cerebellar cortex	*Über Neuesten Untersuchungen* *aus der Nerven- und Hirn-Anatomie* (“About New Investigations on Nerves and Brain”)
1838	Description and illustration of the intracytoplasmic pigment neuromelanin in the substantia nigra	Purkyně, J. E. Neueste Untersuchungen aus der Nerven- und Firnanatomie. *Bericht ü. d. Versammlung deutscher Naturforscher und Ärzte, Prag, Sept. 1837.* Prag, Haase, p. 177–180, 1838.
1847	Neuron theory: concept of the function of the nervous system as a whole	Deiters, O. *Untersuchungen über Gehirn und Rückenmark des Menschen* *und der Säugethiere.* Braunschweig, 1865.
1858	With his coworkers, Purkyně demonstrated a difference between the thickness of the fibers of the posterior sensory roots (smaller diameter) and anterior motor roots (larger diameter)	Purkyně J. E. *Podrobné zprávy o mojích starších i novějších literárních, zvláště přírodnických pracích.* Praha, Živa, p. 36–45, 103–109, 183–189, 242, 246, 1858.

In conclusion, throughout his brief 52 years of life, Purkyně left a legacy extending beyond the medical field—be it cardiology, ophthalmology, and, most remarkably, neurology. The physiologist also made discoveries regarding pharmacology, literature, and forensics science, revealing his curiosity and passion for knowledge. The year of 2025 marks 156 years since his death. However, Purkyně's contributions remain relevant and alive over a century later.
